# Bacterial lipopolysaccharide potentiates type II collagen-induced arthritis in mice

**DOI:** 10.1155/S0962935192000425

**Published:** 1992

**Authors:** Robert G. Caccese, John L. Zimmerman, Richard P. Carlson

**Affiliations:** Inflammation/Bone Metabolism Division Wyeth-Ayerst Research Princeton NJ 08543-8000 USA

## Abstract

Collagen-induced arthritis (CIA) is an immunologically relevant animal model of human rheumatoid arthritis. Studies comparing the disease incidence in genetically susceptible male and female DBA/1LacJ mice demonstrated that under low density/low stress housing conditions, female mice had earlier onset (day 35) and higher disease incidence (25%) than the male mice (17% at day 49) when immunized with bovine type II collagen. A single subcutaneous or intraperitoneal injection of bacterial lipopolysaccharide (LPS) 17–24 days after collagen immunization greatly potentiated this standard CIA model in a dose related manner. 20–40 μg of LPS accelerated the onset of disease from day 35 to day 21 and exacerbated the clinical severity score from 0.27 to 2.00 at day 42. A similar administration of 6 μg of recombinant interleukin-β produced a comparable potentiated CIA model. The acute phase protein, serum amyloid P (SAP), was elevated in the serum at day 26 to 440 μg ml^−1^ for the LPS potentiated CIA mice compared to 65 μg ml^−1^ in the non-potentiated immunized CIA mice. There was a significant correlation (r = 0.78) between SAP levels and disease expression in the LPS treated CIA mice. The rapidity and uniformity of disease expression in this LPS potentiated CIA model will allow more and different drugs to be evaluated with a smaller number of animals.


Mediators of Inflammation, 1, 273–279 (1992)

### Bacterial lipopolysaccharide potentiates type II collagen-induced arthritis in mice

**Robert G. Caccese[CA], John L. Zimmerman and Richard P. Carlson**

Inflammation/Bone Metabolism Division, Wyeth-Ayerst Research, Princeton, NJ 08543-8000, USA

[CA] Corresponding Author

**COLLAGEN-induced arthritis (CIA) is an immunologically relevant animal model of human rheumatoid arthritis. Studies comparing the disease incidence in genetically susceptible male and female DBA/1LacJ mice demonstrated that under low density/low stress housing conditions, female mice had earlier onset (day 35) and higher disease incidence (25%) than the male mice (17% at day 49) when immunized with bovine type II collagen. A single subcutaneous or intraperitoneal injection of bacterial lipopolysaccharide (LPS) 17–24 days after collagen immunization greatly potentiated this standard CIA model in a dose related manner. 20–40 μg of LPS accelerated the onset of disease from day 35 to day 21 and exacerbated the clinical severity score from 0.27 to 2.00 at day 42. A similar administration of 6 μg of recombinant interleukin-1β produced a comparable potentiated CIA model. The acute phase protein, serum amyloid P (SAP), was elevated in the serum at day 26 to 440 μg $ml^{-1}$ for the LPS potentiated CIA mice compared to 65 μg $ml^{-1}$ in the non-potentiated immunized CIA mice. There was a significant correlation ($r = 0.78$) between SAP levels and disease expression in the LPS treated CIA mice. The rapidity and uniformity of disease expression in this LPS potentiated CIA model will allow more and different drugs to be evaluated with a smaller number of animals.**

**Key words:** Acute phase protein, Collagen-induced arthritis, Interleukin-1, Lipopolysaccharide, Serum amyloid P

### Introduction

Collagen-induced arthritis (CIA) is a polyarthritis induced by immunization with native type II collagen in genetically susceptible strains of animals. This model of arthritis has been established in both the rat[1,2] and mouse.[3,4] Its clinical and histopathological manifestations closely resemble those of human rheumatoid arthritis (RA). However, as a pharmacological model for drug discovery, CIA has certain disadvantages. The onset, incidence, and severity of arthritis is highly variable within and among experiments. It has also been reported that male mice are usually more susceptible to CIA than females[5] and that the immune response to a species specific type II collagen (e.g. chick *vs* bovine) varies in different mouse strains.[6] These parameters were investigated using the DBA/1LacJ mouse CIA model under controlled housing conditions.

It has recently been shown that exogenously administered human recombinant interleukin-1 (IL-1) can potentiate the development of CIA.[7,8] Since exogenous lipopolysaccharide (LPS) is known to stimulate endogenous IL-1 release, we tested the effects of LPS on CIA expression. We investigated the dose response effects of LPS on onset, incidence, and severity and also examined the acute phase protein (APP) response in this disease model. These results were compared to the IL-1 potentiated CIA.

### Materials and Methods

*Materials:* Chicken type II collagen from lathyritic chicks was obtained from Genzyme Corporation, Boston, MA. Bovine type II collagen from calf articular cartilage was obtained from Elastin Products Co., Pacific, MO. Bacterial lipopolysaccharide from *Escherichia coli* strain D127:B8 and complete Freund's adjuvant (CFA), strain H37Ra, were obtained from Difco Laboratories, Detroit, MI. Purina Chow, Formula 5015, was obtained from Purina Mills Inc., St. Louis, MO. Recombinant human interleukin-1 beta (rIL-1β) was obtained from Biogen Corp., Cambridge, MA. DBA/1 LacJ mice, 5–6 weeks old, were obtained from the Jackson Laboratory, Bar Harbor, ME.

*Methods:* DBA/1 LacJ mice, 5–6 weeks old, were housed three to a shoebox-type cage equipped with a microisolator filter cap and corncob bedding. Food and water (steam sterilized tap water) were provided *ad libitum*. Cages were changed weekly. After initial caging, care was taken not to introduce new litter mates or otherwise 'mix' the mice. The

© 1992 Rapid Communications of Oxford Ltd animals were kept in a barrier facility used exclusively for these experiments.

At age 8–12 weeks mice were immunized intradermally at the base of the tail with 0.1 ml of an emulsion of type II collagen prepared in the following manner. Type II collagens were solubilized in 0.01 N acetic acid and emulsified with CFA (1:1 v/v). In experiments where a secondary immunization (booster) was administered, the collagen emulsion was injected at day 21 at the same concentration used in the primary immunization. LPS or IL-1 was injected either subcutaneously (s.c.) or intraperitoneally (i.p.) 17–24 days after immunization. Treatment groups consisted of 9–15 mice.

Mice were assessed 3 days after challenge and weekly thereafter for incidence, frequency and severity score defined as follows: incidence is the number of mice having at least one affected paw (inflammation or joint deformity) divided by the total number of mice per group; frequency is the number of affected paws divided by the total number of paws per group; and the severity score is the sum of the individual clinical scores divided by the total number of mice per group.

The clinical score was determined as follows:

| Clinical Score | Description |
|---|---|
| 0.5 | One or more swollen digits |
| 1.0 | Entire paw swollen |
| 2.0 | Deformity observed after inflammation subsides |
| 3.0 | Ankylosis: total loss of joint function in the paw |

At the conclusion of the studies the entire limb was removed above the knee or elbow, fixed in 10% buffered formalin and sent for histological processing and analysis of joint pathology. Evaluations were performed by Research Pathology Services, Inc., New Britain, PA, in a single blind fashion and the degree of arthritis determined in the forepaw (carpal), knee (stifle) and hindpaw (tarsal/metatarsal). Histopathological incidence is defined as the number of joints with at least one lesion divided by the total number of assessed joints per group. Histopathological severity is the sum of the individual joint histopathological scores divided by the number of mice per group. A histopathological score similar to that used by Hom *et al.*[8] was assigned to each joint based on the following criteria:

| Histopathological Score | Description |
|---|---|
| 0 | No alterations |
| 1 | Minimal synovitis, primarily infiltration of mononuclear cells into synovial membrane |
| 2 | Mild synovitis, no cartilage degeneration or bone changes |
| 3 | Moderate synovitis, mild cartilage and bone changes |
| 4 | Marked synovial inflammation, fibroblast proliferation, cartilage damage and periosteal bone proliferation |
| 5 | Severe synovitis, cartilage damage, periosteal bone proliferation and medullary remodelling |

Statistical differences between control and treated groups were determined by the least significant difference test with all possible pairwise comparisons with $p \leq 0.05$ considered significant.

Mice were bled on day 26 or 28 by retro-orbital plexus method. The sera were collected and frozen until assayed for SAP. SAP was quantitated by SDS-PAGE and immunoblotting technique according to the method of Griswold *et al.*[9] The relationship between clinical score and SAP was analysed by linear regression analysis to determine the correlation coefficient.

### Results

*Comparison of arthritic responses in male and female DBA/1LacJ mice using a single injection of chick or bovine type II collagen:* The susceptibility of male and female mice to arthritis using increasing concentrations of collagen to induce arthritis is reported in Table 1. The results showed that in mice housed under well controlled, low stress conditions, susceptibility to arthritis was greater in females than in males. Only 25% of the male mice injected with 100 μg of chick or bovine collagen became arthritic at day 49–60 (Table 1). In contrast, the female mice given chick collagen showed signs of arthritis as early as day 35 and when the mice received 200 μg of bovine type II collagen, arthritis was observed at day 21 (Table 1). Under all conditions, the highest incidence of arthritis achieved was 50%.

*Effect of a collagen booster immunization on arthritis induction in male and female mice:* Mice that were immunized on day 0 and also received a booster injection of collagen on day 21, showed a greatly enhanced level of arthritic incidence in both male and female mice along with a much earlier onset of disease compared to non-boosted mice (Table 2). When the mice were immunized with either chick or bovine collagen, the female mice had an earlier onset of disease and higher levels of incidence and freqency of arthritis at every time point evaluated than the male mice (Table 2). As an example, female mice which received a 50 μg booster of chick collagen reached

**Table 1.** Effect on disease incidence after a single collagen immunization with chick or bovine type II collagen in male and female mice

| Sex | Dose of collagen (μg) | Days of observation | | | | | |
|---|---|---|---|---|---|---|---|
| | | 21 | 28 | 35 | 42 | 49 | 53(F) or 60(M) |
| **Chick type II** | | | | | | | |
| Males | 12.5 | 0[a] | 0 | 0 | 0 | 0 | 0 |
| | 25 | 0 | 0 | 0 | 0 | 0 | 0 |
| | 50 | 0 | 0 | 0 | 0 | 0 | 0 |
| | 100 | 0 | 0 | 0 | 0 | 17 | 25(1.3)[b] |
| Females | 12.5 | 0 | 0 | 0 | 0 | 0 | 0 |
| | 25 | 0 | 0 | 8(1) | 33 | — | 33(1.5) |
| | 50 | 0 | 0 | 8(1) | 17 | — | 42(1.2) |
| | 100 | 0 | 0 | 25(1.3) | 25 | — | 50(1.8) |
| **Bovine type II** | | | | | | | |
| Males | 50 | 0 | 0 | 0 | 0 | 8 | 8(1) |
| | 100 | 0 | 0 | 0 | 0 | 25 | 25(1.6) |
| | 200 | 0 | 0 | 0 | 0 | 0 | 8(1) |
| Females | 50 | 0 | 0 | 0 | 0 | — | 17(1) |
| | 100 | 0 | 0 | 0 | 0 | — | 33(1.25) |
| | 200 | 8 | 8 | 17(1) | 33 | — | 25(2) |

*n* = 10–12 mice/group.
[a] Incidence = number of mice having at least one affected paw divided by the number of mice per group and expressed as a percentage.
[b] Mean number of affected paws per affected mouse.

a 100% level of incidence by day 42 while the highest incidence achieved in the males under the same conditions was 42% at day 56.

*Dose related effects of LPS on CIA in mice:* Female DBA/1LacJ mice were immunized with 25 μg of bovine type II collagen for studies involving LPS. Mice which received 1, 10, or 100 μg of LPS on day 17 produced a dose related increase in disease incidence of 0%, 50% and 83%, respectively, on day 21 (Figure 1). Mice injected on day 21 with PBS, 1 μg LPS, or 10 μg LPS attained 67% incidence level on days 70, 56, and 42, respectively. In another study, 10–100 μg of LPS per mouse was injected s.c. on day 21 to collagen immunized mice. The results shown in Figure 2 indicate a dose related

**Table 2.** Effect on disease incidence after a collagen immunization plus boost[c] (day 21) with chick or bovine type II collagen in male and female mice

| Sex | Dose of collagen (μg) | Days of observation | | | | | |
|---|---|---|---|---|---|---|---|
| | | 28 | 35 | 42 | 49 | 56 | 63 |
| **Chick type II** | | | | | | | |
| Males | 12.5 | 0[a] | 0 | 0 | 8 | 17 | 17(1)[b] |
| | 25 | 0 | 0 | 8 | 17 | 33 | 33(1.25) |
| | 50 | 8 | 18 | 33 | 33 | 42 | 36(1.75) |
| | 100 | 0 | 17 | 25 | 17 | 33 | 33(1.5) |
| Females | 12.5 | 17 | 50(2) | 58 | 75 | 75 | 92(2.2) |
| | 25 | 17 | 33(1.5) | 50 | 58 | 58 | 67(1.6) |
| | 50 | 42 | 70(1.4) | 100 | 100 | 100 | 100(2.9) |
| | 100 | 33 | 50(2) | 50 | 75 | 83 | 83(2.3) |
| **Bovine type II** | | | | | | | |
| Males | 50 | 0 | 0 | 0 | 0 | 0 | 0 |
| | 100 | 17 | 8 | 25 | 17 | 8 | 8(2) |
| | 200 | 17 | 33 | 50 | 67 | 67 | 50(1.7) |
| Females | 50 | 67 | 85(2.2) | 85 | 85 | 77 | 85(2.8) |
| | 100 | 33 | 58(1.3) | 73 | 82 | 91 | 91(2.1) |
| | 200 | 75 | 75(2.6) | 75 | 75 | 83 | 83(2.7) |

*n* = 10–12 mice/group
[a] Incidence = the number of mice having at least one affected paw divided by the number of mice per group and expressed as a percentage.
[b] Mean number of affected paws per affected mouse.
[c] Collagen injected i.d. for the boost was the same as for the primary immunization.

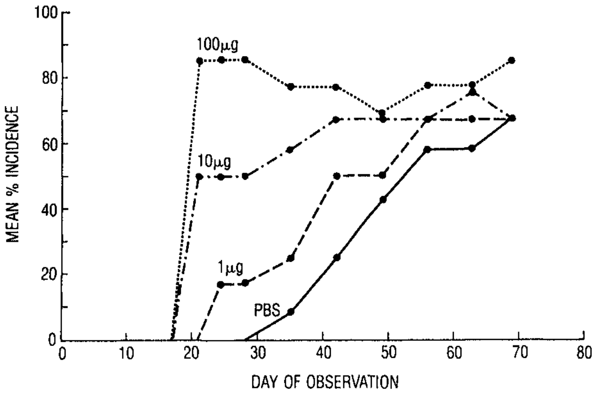

FIG 1. Time course of onset and incidence of CIA in female mice after 1, 10 or 100 µg of LPS per mouse. LPS was administered s.c. on day 17 post collagen immunization. ($n = 12$/group).

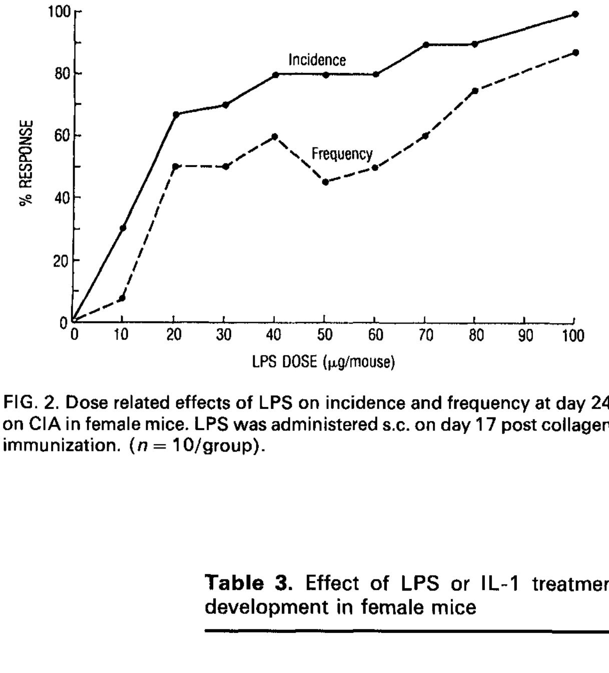

FIG. 2. Dose related effects of LPS on incidence and frequency at day 24 on CIA in female mice. LPS was administered s.c. on day 17 post collagen immunization. ($n = 10$/group).

increase in incidence and frequency on day 24. Similar results were observed when LPS was administered on day 24 (data not shown). It should be noted that CFA emulsion alone (day 0), LPS alone (day 21), or emulsion (day 0) and LPS (day 21) caused no incidence of arthritis to occur in these mice.

*Comparison of LPS potentiated CIA to bovine collagen immunized and boosted mice:* The combined results of studies in which the LPS potentiated CIA was compared to collagen immunized and collagen plus boost immunized CIA showed that female mice sensitized with 50 µg of collagen alone had 0% incidence and 0% frequency on day 28 while those animals immunized with collagen and also boosted with 50 µg of collagen on day 21 had 67% incidence and 21% frequency on day 28. Those mice which were immunized with 25 µg of collagen and given 40 µg of LPS on day 21 had 90% incidence and 70% frequency on day 28.

*Comparison of LPS and IL-1 potentiated CIA:* An experiment was conducted to compare LPS and IL-1 potentiated CIA. Table 3 indicates that 40 µg of LPS injected on day 21 gave an almost equivalent arthritic response to 3 µg of IL-1 injected on day 21 and 22. Using either potentiating agent, the onset of disease occurred on day 25, 3 days post challenge. Clinical incidence was 80% for both LPS and IL-1 treated groups while control (PBS injected) collagen immunized mice showed no incidence on day 25. From day 25 to 42 clinical incidence and severity scores remained elevated and constant for the LPS or IL-1 treated groups while the vehicle (PBS) injected collagen immunized group had

**Table 3.** Effect of LPS or IL-1 treatment on clinical parameters of CIA during disease development in female mice

| Sensitized group | Clinical parameter | Days of observation | | | |
|---|---|---|---|---|---|
| | | 25 | 28 | 35 | 42 |
| 25 µg Bov collagen (day 0) +PBS (day 21) | Incidence (%)[a] | 0 | 20 | 27 | 40 |
| | Frequency (%)[b] | 0 | 5 | 7 | 10 |
| | Severity score[c] | 0 | 0.10 | 0.17 | 0.27 |
| 25 µg Bov collagen (day 0) +40 µg LPS (day 21) | Incidence (%) | 80 | 87 | 73 | 80 |
| | Frequency (%) | 48 | 48 | 45 | 52 |
| | Severity score | 1.86 | 1.80 | 1.60 | 2.00 |
| 25 µg Bov collagen (day 0) +3 µg IL-1 (day 21 & 22) | Incidence (%) | 80 | 80 | 80 | 80 |
| | Frequency (%) | 65 | 67 | 65 | 65 |
| | Severity score | 2.43 | 2.43 | 2.50 | 2.50 |

$n = 15$ mice/group.

[a] Incidence = the number of mice having at least one affected paw divided by the number of mice per group and expressed as a percentage.

[b] Frequency = the number of affected paws divided by the total number of paws per group and expressed as a percentage.

[c] Severity score = the sum of the individual clinical scores (see Methods) divided by the total number of mice per group.

**Table 4.** Effect of LPS or IL-1 treatment on histopathological parameters of CIA at day 42 in female mice

| Sensitized group | Histopathological parameter | |
|---|---|---|
| | Incidence (%)[a] | Mean Severity score[b] |
| 25 μg Bovine collagen (day 0) +PBS (day 21) | 44 | 2.07 |
| 25 μg Bovine collagen (day 0) +40 μg LPS (day 21) | 64 | 4.60* |
| 25 μg Bovine collagen (day 0) +3 μg IL-1 (day 21 & 22) | 62 | 4.93* |

$n = 15$ mice/group.
* $p \leq 0.05$, compared to immunized mice without LPS or IL-1 treatment.
[a] Incidence = the number of joints with at least one lesion (e.g. synovitis, cell infiltration, cartilage degeneration, bone changes) divided by the total number of assessed joints per group and expressed as a percentage.
[b] Severity score = the sum of the individual joint histopathological scores (see Methods) divided by the number of mice per group.

increasing but low clinical incidence and severity scores during the same period (Table 3).

On day 42 the limbs of the mice from the above experiment were removed and processed for histopathological evaluation. Although the degree of synovitis, cellular infiltration, cartilage degradation, and aberrant bone proliferation varies within as well as among treatment groups, the LPS and IL-1 potentiated groups produced similar histopathological severity scores (4.6 and 4.9, respectively) at day 42 while the control immunized group exhibited a severity score (2.1) less than 50% of the potentiated CIA treated groups (Table 4).

*Comparison of SAP levels in mice with LPS or IL-1 potentiated CIA.* The acute phase protein, serum amyloid P (SAP), was measured on the 26th day after collagen immunization (Table 5), 5 days after the LPS or IL-1 injection in the female DBA/1LacJ mice. The SAP levels from the LPS or IL-1 potentiated CIA mice were approximately 10–14 times higher than the naive or emulsion injected animals and 5–7 times higher than the collagen immunized non-arthritic mice. The LPS potentiated immunized mice had SAP levels about twice as high as their control, nonimmunized but LPS injected mice (441 μg $ml^{-1}$ and 194 μg $ml^{-1}$, respectively). In Fig. 3A and 3B, SAP levels and clinical scores on day 28 from LPS potentiated or IL-1 potentiated CIA mice showed significant correlations of $r = 0.78$ and $r = 0.85$, respectively.

### Discussion

In these experiments, we have shown that under low density/low stress housing conditions, genetically susceptible DBA/1LacJ female mice exhibit earlier onset of disease and higher disease incidence when using either chick or bovine type II collagen immunization than male mice in the traditional CIA model. The higher female than male arthritic response is contrary to what has been reported.[5] The lower CIA response in male mice housed under

**Table 5.** Effect of LPS or IL-1 treatment on SAP levels in collagen immunized and non-immunized female mice at day 26

| Sensitized group | Clinical Severity score[a] | SAP level (μg $ml^{-1}$ serum) |
|---|---|---|
| PBS, day 21 | 0.00 | 31 ± 3[b] |
| Emulsion, day 0; & PBS, day 21 | 0.00 | 36 ± 3 |
| LPS only (40 μg), day 21 | 0.00 | 194 ± 38 |
| Collagen (25 μg), day 0; & PBS day 21 | 0.00 | 65 ± 8 |
| Collagen (25 μg), day 0; & LPS (40 μg), day 21 | 3.67 | 441 ± 73* |
| Collagen (25 μg), day 0; & IL-1 (2 μg), day 21, 22, 23 | 3.00 | 352 ± 133* |

$n = 15$ mice/group.
* $p < 0.05$ compared to control mice (emulsion + PBS).
[a] Clinical Severity score = sum of the individual scores divided by the number of mice per group (see Methods for further detail).
[b] Mean ± SEM.

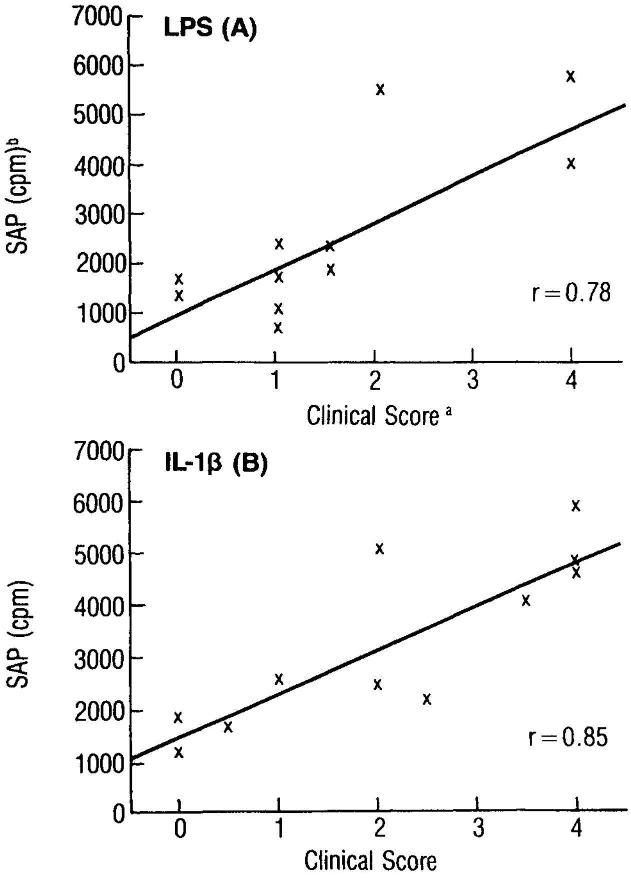

FIG. 3. Positive correlation of clinical scores compared to SAP levels on day 28, 7 days after CIA mice were injected i.p. with LPS (A) or IL-1β (B).
[a] Clinical score is defined in Methods.
[b] SAP levels are expressed as CPM with serum diluted 1:50 for assay (see Methods for details).

controlled low density conditions may be due to the lack of the male fighting syndrome, which reduces stress in these animals to a minimum. It is also possible that the usual male fighting could have led to subclinical infections and their associated inflammatory mediators which could exacerbate their CIA. Our results with the male CIA mice support using female mice in the CIA as a model of human RA, since it is known that females have a higher incidence of RA than males.[10]

Other investigators have shown that rIL-1β can increase the incidence and potentiate CIA in mice.[7,8] This cytokine is known to stimulate the proliferation of T cells, induce the release of lymphokines, and activate neutrophils, synoviocytes, chondrocytes, osteoblasts and osteoclasts to release inflammatory mediators, such as eicosanoids and collagenases.[11] These combined factors may play a role in the CIA arthritic disease model. We have demonstrated that LPS, possibly by inducing endogenous IL-1 release, can also potentiate CIA in a dose dependent manner. LPS accelerates the onset and increases the severity of CIA such that marked inflammation is observed just 72 h after LPS or IL-1 administration in the collagen immunized mice. The disease progression in non-LPS treated mice occurred slowly with variable onset and gradual increase of incidence from day 28 to day 60–70. The histopathological severity score for LPS treated mice at day 42 was 200% greater than the severity score of the non-potentiated CIA mouse model. These histopathological results indicate that the LPS stimulated arthritic disease at day 42 resembles an advanced stage of disease seen much later in the normal CIA model. This is similar to what has been reported when IL-1 is used as the potentiating agent.[12] Therefore, it appears that the collagen immunized mice (i.e. animals with serum anti-type II collagen antibodies) are 'primed' to develop arthritis, and that IL-1 or LPS can be the triggering mechanism for a rapid induction of arthritis development.

The APP, C-reactive protein (CRP), is measured in humans with chronic arthritic disease.[13] We investigated the APP response in this disease model using a major murine acute phase reactant, serum amyloid P, which is structurally related to CRP and has 70% amino acid homology.[14] Since it is known that LPS and IL-1 can elevate APP *in vitro* and *in vivo*,[15–18] our results are similar to those reported by others[19,20] using the non-LPS potentiated CIA model. There was a significant correlation between clinical severity score and SAP concentration. The correlation for the IL-1 injected animals was similar to the LPS treated mice. This potentiated CIA model showed that the acute phase response is associated with severity of disease and may be used as an index of arthritis. With the appearance of arthritis (paw inflammation) the SAP levels were increased seven-fold compared to the immunized non-potentiated mice who had no inflammation. This difference in elevated SAP levels is probably a result of cellular influx and cytokine release.[11] SAP level measurement, as a representative of acute phase response, may be an indicator of different mechanisms of action for different classes of drugs tested in this model.[19]

Additional experiments are planned to further characterize this potentiated CIA model. Areas to be investigated include: (1) does LPS administration have an effect on type II collagen antibody levels; (2) does passive transfer of arthritis last longer after LPS treatment; (3) does LPS alter the function of T cells from blood, lymph nodes and spleens of type II collagen sensitized mice; (4) do SAP levels correlate with the progression and severity of disease at later time periods; and (5) does LPS affect the late stage (day 70–90) parameters such as ankylosis and bone changes in CIA mice.

The LPS potentiated CIA mouse model has many advantages for use in pharmacological studies. It reduces the latent period required for the development of arthritis, while producing early high levels of clinical inflammation and histopathological incidence of arthritis in a synchronized disease state. In addition, this model will allow more drugs to be tested with a smaller number of animals and to evaluate different classes of drugs (e.g. anti-inflammatory, immunomodulatory, immunosuppressive, and remission inducing drugs) in both prophylactic and therapeutic regimens.[12,21,22]

### References

1. Trentham DE, Townes AS, Kang AH. Autoimmunity to type II collagen: an experimental model of arthritis. *J Exp Med* 1977; **146:** 857–868.
2. Stuart JM, Gremer MA, Kang AH, Townes AS. Collagen-induced arthritis in rats: evaluation of early immunologic events. *Arthritis Rheum* 1979; **22:** 1344–1351.
3. Courtenay JS, Dallman MJ, Dayan AD, Martin A, Mosedale B. Immunization against heterologous type II collagen-induced arthritis in mice. *Nature* 1980; **2843:** 666–668.
4. Wooley PH, Luthra HS, Stuart JM, David CS. Type II collagen-induced arthritis in mice. I. Major histocompatibility complex (I-region) linkage and antibody correlates. *J Exp Med* 1981; **154:** 688–700.
5. Wooley PH, Chapedelaine JM. Immunogenetics of collagen-induced arthritis. *CRC Crit Rev Immunol* 1987; **8:** 1–22.
6. Wooley PH, Dillon AM, Luthro HS, Stuart JM, David CS. Genetic control of type II collagen-induced arthritis in mice: factors influencing disease susceptibility and evidence for multiple MHC-associated gene control. *Transplant Proc* 1983; **15:** 180–185.
7. Killar LM, Dunn CJ. Interleukin-1 potentiates the development of collagen arthritis in mice. *Clin Sci* 1989; **76:** 535–538.
8. Hom JT, Bendele AM, Carlson DG. *In vivo* administration with IL-1 accelerates the development of collagen-induced arthritis in mice. *J Immunol* 1988; **141:** 834–841.
9. Griswold DE, Hillegass LM, Antell L, Shotzman A, Hanna N. Quantitative western blot assay for measurement of the murine acute phase reactant, serum amyloid P component. *J Immunol Meth* 1986; **91:** 163–168.
10. Gilliland BC. Rheumatoid Arthritis: A model of chronic inflammation. *Arzneim-Forsch/Drug Res* 1989; **39:** 952–955.
11. Dinarello CA. Interleukin-1 and interleukin-1 antagonism. *Blood* 1991; **77:** 1627–1652.
12. Hom JT, Gliszczynki VL, Cole HW, Bendele AM. Interleukin-1 mediated acceleration of type II collagen-induced arthritis: effects of anti-inflammatory or anti-arthritic drugs. *Agents Actions* 1991; **33:** 300–309.
13. Whicher JT, Thompson D, Billingham MEJ, Kitchen EA. *Acute phase proteins. In* Chang JY, Lewis AJ, eds. *Pharmacological methods in the control of inflammation*, New York: Alan R. Liss, Inc. 1989; **5:** 101–128.
14. Pepys MB, Baltz ML, deBeer FC, *et al.* Biology of serum amyloid P component. *Ann NY Acad Sci* 1982; **389:** 141–211.
15. Le PT, Muller MT, Mortensen RF. Acute phase reactants of mice: I. Isolation of serum amyloid P-component (SAP) and its induction by a monokine. *J Immunol* 1982; **129:** 665–672.
16. Le PT, Mortensen RF. *In vitro* induction of hepatocyte synthesis of the acute phase reactant mouse serum amyloid P-component by macrophages and IL-1. *J Leukoc Biol* 1984; **35:** 587–603.
17. Mortensen RF, Beisel K. Zeleznik NJ, Le PT. Acute phase reactants of mice: II. Strain dependence of serum amyloid P-component (SAP) levels and response to inflammation. *J Immunol* 1983; **130:** 885–889.
18. Mortensen RF, Shapiro J, Lin BF, Douches S, Neta R. Interaction of recombinant IL-1 and recombinant tumour necrosis factor in the induction of mouse acute phase proteins. *J Immunol* 1988; **140:** 2260–2266.
19. Griswald DE, Hillegrass LM, Meunier PC, DiMartino MJ, Hanna N. Effect of inhibitors of eicosanoid metabolism in murine collagen-induced arthritis. *Arthritis Rheum* 1988; **30:** 1406–1412.
20. Bliven ML, Wooley PH, Pepys MB, Otterness IG. Murine type II collagen arthritis: association of an acute-phase response with clinical course. *Arthritis Rheum* 1986; **29:** 1131–1137.
21. Adams LM, Caccese RG, Cummons TA, Sehgal SN, Chang JY. Effect of the immunosuppressants rapamycin (RAPA) and cyclosporin A (CsA) on Collagen-Induced Arthritis (CIA) in mice. *FASEB J* 1990; **4:** A358.
22. Caccese RG, Cummons TA, Chang JY, Adams LM. Effect of the 5-lipoxygenase (5-LO) inhibitor, WY-50,295 tromethamine, and indomethacin on lipopolysaccharide (LPS)-challenged collagen-induced arthritis (CIA) in mice. *FASEB J* 1991; **5:** A511.

ACKNOWLEDGEMENTS. We thank Terri A. Cummons for her excellent technical assistance in the care of the animals and Maria Tulanowski and Jessica Noll for the typing of this manuscript.

**Received 12 May 1992; accepted in revised form 12 June 1992**
